# Human factors methods for designing safe health information technology: what do the experts think?

**DOI:** 10.1093/jamia/ocag106

**Published:** 2026-06-29

**Authors:** Selvana Awad, Rachel Begg, Thomas Loveday, Andrew Baillie, Melissa T Baysari

**Affiliations:** The University of Sydney, Australia; eHealth NSW, Australia; eHealth NSW, Australia; eHealth NSW, Australia; The University of Sydney, Australia; The University of Sydney, Australia

**Keywords:** human factors, patient safety, health information technology

## Abstract

**Objective:**

Human Factors (HF) methods have potential to ensure the design of health information technology (HIT) is safe and usable, but limited research has examined what HF methods have been applied or could be applied to HIT. This study aimed to identify key HF and safety analysis methods used and/or recommended by HF experts working in healthcare or non-healthcare industries that may be suitable for supporting the design, redesign and configuration of HIT.

**Materials and Methods:**

We ran semi-structured interviews with 21 HF experts working across health and non-health industries to identify key HF and safety analysis methods used and/or recommended for supporting the design, redesign and configuration of HITs.

**Results:**

Forty-seven HF methods across 11 method categories were reported by HF experts. Non-health participants reported a wider range of Human Error Identification and Risk Assessment Methods and were the only cohort to report workload assessment methods. Both health and non-health participants described their approaches for selecting methods and recommended that methods be applied flexibly.

**Discussion:**

This study highlights the need to uplift HF capability by teaching methods to system designers, particularly where access to HF experts is limited and undertaking efforts to learn from HF practice in safety-critical industries outside of healthcare.

**Conclusion:**

Further work is required to integrate HF methods and approaches, especially those focused on safety, into the work of HIT designers.

## Introduction

Health information technology (HIT) includes technologies that support the process, storage, and exchange of health information such as electronic medical records, electronic medication management systems (EMMS), health information exchange systems, patient portals, and virtual care platforms.^1^^,^^2^ Poorly designed HIT can result in usability issues and in turn, “use errors,” which are actions performed by the user that are not intended by the manufacturer or designer of the system.^3–5^ Examples of interface design flaws that can contribute to use errors include too many options to choose from, misuse of colors that do not comply with color conventions, or designs that require users to navigate to and remember information from different screens to perform a task.^1^^,^^2^^,^^4–7^ In healthcare, these can both compromise system safety and result in patient harm.^4^^,^^8–11^ For example, poorly designed HITs have been shown to result in inefficient care provision, as tasks take longer to complete, decreased user satisfaction, and sub-optimal system use (eg, workarounds).^9–11^ In addition to interface design, the safety of health IT is also influenced by complex sociotechnical factors outside of the IT system such as clinical practices, organizational policies, staffing/resources, and other dynamic contextual factors at play within the local environment and hospital ecosystem in which the technology is implemented.^12–15^ Good HF design encompasses the design of all these elements in the work system. The work system consists of the people, tasks, tools/technologies, the physical environment, and organizational conditions that determine how work is performed and the outcomes of the work.^10–15^

In the context of HIT system design, Human Factors (HF) or ergonomics can contribute to “safety by design” by designing the system to help users do the right thing and making it difficult to do the wrong thing.^4^^,^^16–18^ While safety-critical industries such as aviation, manufacturing, transportation, nuclear power, and the military have seen the systematic application of HF through HF integration, the application of HF in healthcare, including in HIT, is more recent.^4^^,^^6^ The limited use of HF and safety analysis methods, possibly due to limited HF workforce and expertise in healthcare, to proactively identify, assess and address hazards and safety critical issues by designing them out has been documented in the literature.^19^ Most applications of HF in the HIT context have relied on methods related to human-computer interaction, a subarea of HF focused on issues of usability, that is, how the interface’s design affects user experience, and how users effectively and efficiently use the system.^19–21^ For example, a recent systematic review identified that the HF methods most frequently used to support the design and redesign of EMMS (a type of HIT used for medication management) are prototyping, usability testing, participant surveys/questionnaires, and interviews,^20^ with limited utilization of more safety-oriented HF and workload assessment methods.^20^

To complement the limited information that exists in the literature regarding HF and safety analysis methods that have been applied to support the design, redesign, and configuration of HIT, this current study sought to collect data around the application of HF and safety analysis methods in this context from an alternative source: practicing HF experts. Given the low application of HF methods in healthcare, to capture a wider range of HF methods, we sought this advice from both HF experts working in healthcare and non-healthcare industries. This study is one of the first to interview HF experts across multiple industries on HF methods that can support HIT design and document which methods may be applied, as well as practical considerations on how methods are selected and applied. No published studies have sought to capture such expert advice with regards to the selection, application and evaluation of HF and safety analysis methods to support safe HIT design and redesign.

## Objectives

This study aimed to identify key HF and safety analysis methods used and/or recommended by HF experts working in healthcare or non-healthcare industries that may be suitable for supporting the design, redesign, and configuration of HIT. The study also aimed to compare responses from HF experts working in healthcare with those not working in healthcare.

## Materials and methods

### Design

This was a qualitative study which comprised semi-structured interviews with HF experts working across health and non-health industries in academic and/or practitioner roles. For the purpose of this study, an HF expert was considered someone with a relevant degree (eg, ergonomics, HF engineering) and who was actively using their HF expertise. Ethics approval was obtained from the University of Sydney’s Human Research Ethics Committee. The Consolidated Criteria for Reporting Qualitative Research Checklist was used to guide manuscript preparation.^22^

### Recruitment

Participants were recruited through a purposive sampling method in conjunction with snowball sampling over a 4-month period (December 2022-March 2023). This involved advertising the study through national HF societies and relevant working groups, recruitment at an international HF and patient safety conference through networking and word-of-mouth approaches, consultation with HF experts and contacts to recommend other practitioners, and a review of HF textbooks and literature to identify HF authors who could be interviewed. Forty-one participants were directly invited to participate; one declined and 19 did not respond. Participants who did not respond were followed up once after initial contact. Recruitment for each group (health and non-health) continued until thematic saturation was reached within each group. Suitable participants interested in participating, including those identified by the investigators, were invited to take part in the study via email. All participants provided informed consent and agreed to be audio-recorded.

### Data collection

All interviews were conducted by a clinical informatics professional with expertise in design, HF, and safety and quality (S.A.). The interviews were primarily conducted by videoconferencing except one which occurred face to face. They explored which HF methods participants had used in the context of IT, which they have used and/or would recommend for designing, redesigning and configuring HIT, and how they apply and select methods. Questions used to guide the semi-structured interviews (Supplementary Material Appendix A), some of which were informed by The Consolidated Framework for Implementation Research,^23^ were developed by S.A. and a HF expert (M.B.), and reviewed by other members of the research team with HF (T.L. and R.B.) and implementation science expertise (A.B.). These questions served as a guide only and some aspects were explored in more or less depth as interviews continued and particular themes and concepts emerged. If participants had not previously applied methods to HIT design, redesign, or configuration, they were provided with a brief explanation of HIT such as EMMS and asked to recommend appropriate methods for this use case. All interviews were audio-recorded and transcribed verbatim.

### Data analysis

De-identified content from the interview transcripts was independently analyzed by 2 investigators with expertise in HF (S.A. and R.B.) using a general inductive approach. Each investigator independently coded the first 5 interviews, and then met to discuss findings and reach consensus on a high-level framework to support the documentation of themes. For the remainder of the interviews, each investigator continued to independently assign codes to text and map these codes to the agreed themes using the framework (Supplementary Material Appendix B). At the end of this process, the researchers met together with a third researcher, a Professor of HF with extensive experience in qualitative research, to discuss codes and themes, and any further discrepancies until consensus was reached. Overall, the 2 investigators were generally consistent in their identification of themes. Disagreements were minor and were resolved via a discussion between the 3 researchers.

## Results

### Participant demographics

A total of 21 participants took part, and interviews lasted on average 52 minutes (range 39-103 minutes). Table 1 describes the demographics of the 21 participants with respect to their industry and roles. Overall, 14 of 21 (66.7%) worked in the health industry (eg, hospitals or university health research) while 7 (33.3%) worked in non-health industries (eg, aviation and transport). Eight were from Australia, 4 from the United States, 3 from the Netherlands, 2 from France, 1 from Israel, 1 from the United Kingdom, 1 from Italy, and 1 from Denmark.

**Table 1.** Characteristics of participants who participated in interviews by core industry and roles.

Core industry	Core role	Counts	% within industry category	% within total participants
Health (*n* = 14)	Academic	9	64.3 %	42.9 %
	Practitioner	2	14.3 %	9.5 %
	Academic/Practitioner	3	21.4 %	14.3 %
*Total*		14		66.7%
Non-health (*n* = 7)	Academic	2	28.6 %	9.5 %
	Practitioner	5	71.4 %	23.8 %
	Academic/Practitioner	0	0.0 %	0.0 %
* Total*		*7*		*33.3%*
*Total*		21		100%

There were no differences in methods or approaches reported by participants when asked about IT systems in general or HIT in particular, so the results for these were grouped and are presented together below.

### Methods used and/or recommended by human factors experts to design, redesign, or configure IT

Table 2 describes methods used and/or recommended by both health and non-health participants for the design, redesign, or configuration of IT, grouped by method categories, and their purposes. Across all participants, 47 methods were reported which fell into 11 broad method categories. In general, health and non-health participants reported the same method categories, however, there appeared to be some differences in the individual methods recommended by the 2 groups. For example, non-health participants cited a wider range of Human Error Identification and Risk Assessment Methods than health participants. Only non-health participants cited methods related to workload and other forms of testing (eg, site acceptance testing), and only health participants cited process charting methods and safety probes.

**Table 2.** Methods and their purposes recommended by HF experts.

**1. Method Category: Data Collection Methods**	
*Examples of methods/tools cited*	*Key purpose/s of methods*
1.1 Observations (H, NH) 1.2 Interviews (H, NH)	To understand the work system/context/practices/tasks To understand how users interact with the system To understand the problem at hand and issues (eg, usability and safety issues, use errors, workarounds)
**2. Method Category: Process Charting**	
*Examples of methods cited*	*Key purpose/s of methods*
2.1 Process charting (H) 2.2 Context mapping (H) 2.3 Patient journey mapping (H)	To understand the work system/context/practices/tasks To understand the problem at hand and issues (eg, usability and safety issues, use errors, workarounds)
**3. Method Category: Task Analysis**	
*Examples of methods cited*	*Key purpose/s of methods*
3.1 CTA (H, NH) 3.2 HTA (H, NH) 3.3 Critical decision method (H) 3.4 Critical incident technique (H) 3.5 Cognitive walkthrough (H, NH)	To understand the work system/context/practices/tasks To identify and address usability and safety issues To understand how users interact with the system To understand cognition behind real-life actions To understand systemic contributors to failure events or problems within a process
**4. Method Category: Analysis of Available Data**	
*Examples of methods cited*	*Key purpose/s of methods*
4.1 Incident analysis (H, NH) 4.2 Analysis of click data (H) 4.3 Review of databases with relevant information (eg, MAUDE) (H) 4.4 Audits (NH)	To feed safety information and regulatory requirements into the procurement/design/redesign process To understand how users interact with the system To support the identification of design issues that should be prioritized for redesign
**5. Method Category: Interface Analysis**	
*Examples of methods cited*	*Key purpose/s of methods*
5.1 Link analysis (H, NH) 5.2 Usability testing (formative and summative) (H, NH) 5.3 Heuristic evaluation (H, NH) 5.4 Usability/design/human factors principles or checklists (H, NH) 5.5 Eye tracking (H) 5.6 Face reading (H) 5.7 A/B Testing (H) 5.8 Surveys 5.8.1 SUS (H) 5.8.2 PSSUQ (H) 5.8.3 Usability, satisfaction and performance-based surveys (NH)	To identify and address usability and safety issues To test and iteratively enhance prototypes To understand user experience and interactions with the system To evaluate system usability
**6. Method Category: Human Error Identification and Risk Assessment Methods**	
*Examples of methods cited*	*Key purpose/s of methods*
6.1 FRAM (H) 6.2 ACCIMap maps (H, NH) 6.3 FMEA (H) 6.4 uFMEA (H) 6.5 HFMEA (H) 6.6 Human factors risk assessments (NH) 6.7 STAMP (NH) 6.8 NET-HARMS (NH) 6.9 STPA (NH) 6.10 SHERPA (NH) 6.11 HEART (NH) 6.12 Human Error HAZOP (NH) 6.13 O&SHA Workshops (NH)	To understand the work system/context/practices/tasks To understand the problem at hand and issues (eg, usability and safety issues, use errors, workarounds) To identify systemic contributors to failure events or incidents/accidents, hazard causes, and appropriate controls To identify and address usability and safety issues To understand the severity of identified problems To identify what can go wrong (ie, human errors and risks that can emerge) and potential error causing conditions to address vulnerabilities in a design/process/task
**7. Method Category: Design Methods**	
*Examples of methods cited*	*Key purpose/s of methods*
7.1 Prototyping/wireframing (H, NH) 7.2 Storyboards (NH) 7.3 Participatory design (H, NH) 7.4 Co-design (H) 7.5 Workshops/focus groups/meetings/design sessions (H, NH)	To generate, test and iteratively enhance a potential design To identify and address usability and safety issues To identify requirements
**8. Method Category: Requirements Analysis**	
*Examples of methods cited*	*Key purpose of methods*
8.1 Requirements analysis (H, NH)	To identify requirements including those related to usability and safety
**9. Method Category: Safety Probes**	
*Examples of methods cited*	*Key purpose of methods*
9.1 Safety probes (H)	To test if the design is helping the user identify safety problems, for example, alerts for medication safety concerns
**10. Method Category: Other Testing**	
*Examples of methods cited*	*Key purpose of methods*
10.1 SAT (NH) 10.2 FAT (NH)	To identify any final bug fixes, major issues or usability problems prior to release
**11. Method Category: Workload Assessments**	
*Examples of methods cited*	*Key purpose of methods*
11.1 NASA-TLX (NH) 11.2 Observations, interviews and data analysis of workload (NH) 11.3 Cognitive workload assessments/testing (NH)	To understand the impact of the system and system changes on workload

A/B, A refers to control, B refers to variation; CTA, cognitive task analysis; FAT, factory acceptance testing; FMEA, failure modes and effects analysis; FRAM, functional resonance analysis method; H, health participant; HAZOP, hazard and operability study; HEART, the human error assessment and reduction technique; HFMEA, healthcare failure mode and effect analysis; HTA, hierarchical task analysis; MAUDE, manufacturer and user facility device experience; NASA-TLX, NASA task load index; NET-HARMS, networked hazard analysis and risk management system; NH, non-health participant; O&SHA, operating & safety hazard analysis workshops; PSSUQ, post-study system usability questionnaire; SAT, site acceptance testing; SHERPA, the systematic human error reduction and prediction approach; STAMP, the systems theory accident modeling and process; STPA, system-theoretic process analysis; SUS, system usability scale; uFMEA, use failure mode effects analysis.

### How human factors experts selected methods to support the design, redesign, or configuration of IT

Both health and non-health participants described several factors that they consider when selecting HF methods, including the context of application, the effectiveness of the method in achieving the aim of its application, and the feasibility of applying the method. A full list of factors is available in Supplementary Material Appendix C.

Participants explained that their decision-making process around method selection may sometimes be guided by a framework or approach: “*it’s all about the framework of methods, it’s not about the single method.*”[NH1] For example, considering which HF domain areas/constructs are required for the project or system (eg, usability, safety, workload, situational awareness, decision making) and gaps/limitations of each method can inform which method combinations may provide appropriate coverage based on the need. Both health and non-health participants described the use of systems-based approaches to understand the work system, how users interact with an IT system within a complex, sociotechnical environment and how different system elements impact each other. Examples provided by health participants included the Systems Engineering Initiative for Patient Safety model and a work system analysis, and non-health participants cited the Event Analysis of Systemic Teamwork, Cognitive Work Analysis/Work Domain Analysis (WDA).

A health participant also described the use of a conceptual framework or approach involving micro-, meso-, and macro-levels to guide method selection as reliance on commonly used methods such as usability testing may result in identifying problems only from a linear micro perspective: “*there’s going be interplays [between] all of these three levels…micro, meso, and the macro, and they will all have implications on the design of that system, and how linear it needs to be or how flexible it needs to be…. But I think it’s tempting sometimes to look at just the safety critical problems from a very linear micro perspective*.” [H7]

### How human factors experts applied methods to support the design, redesign, or configuration of IT

Participants described how they would apply methods. In general, there was more variability in how HF was applied by those working in health while the non-health group was more homogenous in their description of HF application.

#### Understanding the work system and problem

Both the health and non-health groups indicated that as a first step, some core methods should be used to assist with understanding the work context, system, and the problem at hand: “*we have some methods… that are of course needed in terms of the first point is understanding the work situation more.*” Examples of methods suggested for this first step by the health group included interviewing, observations, process mapping, and heuristic or usability evaluation: “*what we always do is an elaborate context study…most of the time, it’s with interviewing and observations, and just go into the workplace, interviewing people about what they do, and what they use in their environment.*” Methods suggested by the non-health group included methods that support an early HF analysis (eg, reviewing available documentation and consultation with end users), gathering of user needs/requirements, WDA and task analysis (eg, hierarchical task analysis): “*one key thing is understanding the tasks. So whether that’s through a hierarchical task analysis, or something like that, but also understanding the interactions between tasks is nice…what the flow is*” [NH7] and “*hierarchy of task analysis… This steps out all the processes and all the actors, and gives us a good overview of…communication interaction perspectives.*” [NH2]

While the health group described the need to understand the problem and understand the work context as an initial step, the non-health group generally described this process as an early HF analysis (EHFA), a term which was not used by the health group. Non-health participants described an EHFA as an activity which helps identify who uses the system, how it operates, what the goals of the system are, the overall work context, safety critical issues (including safety and HF risks) associated with the system that may require prioritized focus and HF activities that will need to be undertaken during the project lifecycle: “*the first thing we would do is …an early human factors analysis…this is basically where we would meet with subject matter experts [SMEs], control operators, operational experts to get the context of use of the system*” [NH2] and “*early human factors analysis is the first thing always.*” [NH5]

#### Methods dependent on whether IT was being designed or redesigned

Non-health participants described that the exact methods applied as part of a HF activities could differ depending on whether an IT system was being newly designed or an existing system was being redesigned. For example, “*if you were designing [a system] from scratch, you would ask each [user] what they needed from it…based on the tasks that they’re performing. If we were redesigning an existing system, we would start with looking at incidents or previous incidents… to understand what the baseline performance was…how many people can do it right now, before we improve it…if it’s an existing system, then you should be able to collect quite a lot of data about what works well, and what’s not working well now. And then those can become part of the user requirements.*” [NH3]

#### Flexibility in method application

In general, there was more homogeneity in how practitioners working in non-health sectors described the structured application of HF through human factors integration (HFI) compared to health participants. Similar to EHFA, the term “human factors integration” was only mentioned by non-health participants to describe how methods would be applied within a given project following completion of an EHFA: “*we have what’s called the Human Factors integration process… there’s an Australian standard …which…talks about how human factors should be involved in projects and where it should be involved and that’s what we follow.*” [NH2]

Both health and non-health participants described the need to apply methods flexibly and adapt them for the project and context rather than applying them in a purist way: “*I’ve never once done the purist form in my career on any of these methods*” [NH2] and “*that’s one of the good things about HF methods is that they are very flexible…you can apply parts of them, all of them, or combinations of them.*” [NH6] They described the need to balance robustness with pragmatism, for example by applying methods in a way that provides sufficient data for the need and knowing when to stop collecting data: “*don’t be afraid to use methods that seem less robust, because they actually sometimes provide the better, more pragmatic outcomes*” and “*there’s another rule that you must have with these HF analyses and it’s a stopping rule. How much is enough? Do we have enough data for the risk that we’re considering? That’s really important.*” [NH4] Participants mentioned that time, resourcing and other project constraints may warrant adaptable application of methods to ensure efficiency and feasibility: “*how much value that method is going to apply as well. So how much? How much value would a detailed task analysis provide over a high level task analysis? For example, do you need to go down to the X granular level or can you stay at the high level to get what you need? So it’s kind of those three factors: value, time, money.*” [NH3]

#### Role of human factors expertise

Both health and non-health participants advocated for the early (eg, during procurement) and ongoing involvement of HF experts in projects to ensure system selection, design and redesign are supported by appropriate HF activities: “*probably the biggest takeaway is that as a human factors practitioner, you add the most value to any project at the beginning…involve early in the initial phases before products is conceived, and identify the Human Factors work required at that beginning.*” [NH3] Due to challenges with accessing human factors expertise particularly in the health context, participants from both groups advocated for building HF capability in healthcare, for example by training clinicians, system designers and other relevant personnel to apply HF thinking, and felt that some HF activities could be undertaken by trained non-HF experts: “*we need to have robust application of human factors in the hands of reasonably experienced, but still not expert, clinicians*” [H7] and “*I’m a big advocate that human factors isn’t a locked out or exclusive discipline. Anybody can learn to use the methods and the techniques that we use, with the right sort of guidance and the amount of time to actually learn and sit there and apply them.*” [NH3]

### Guidance for practical application based on synthesized findings

Figure 1 provides a consolidated, synthesized representation of results in the form of practical guidance for those selecting and applying HF methods to HIT design and redesign.

**Figure 1.**
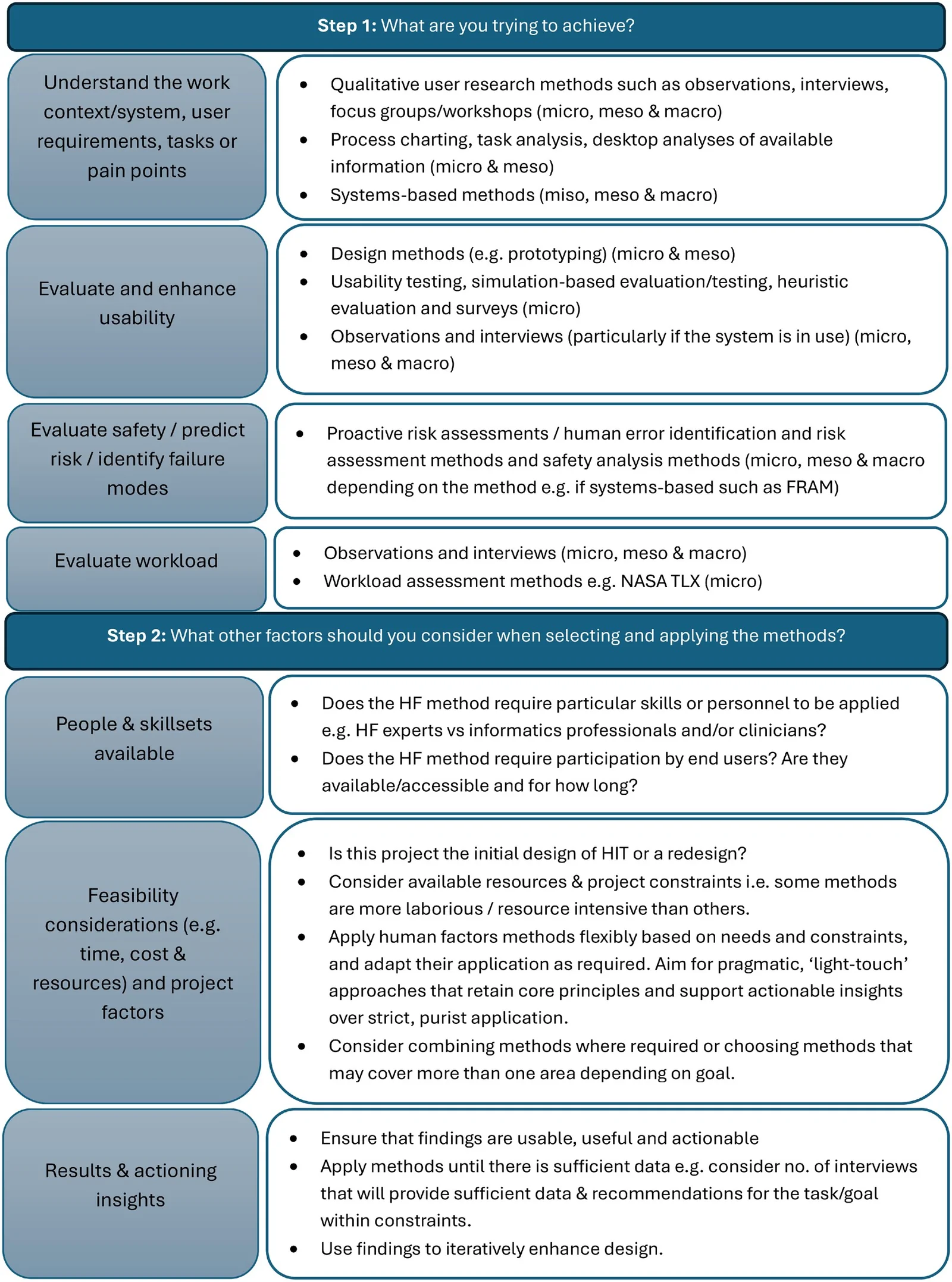
Practical representation of results for those selecting and applying HF methods to HIT design and redesign.

## Discussion

This study, one of the first to interview HF experts across multiple industries on HF methods that can support HIT design, identified 47 HF and safety analysis methods and also uncovered the approaches HF experts take to method selection and application. Non-health participants reported a wider range of Human Error Identification and Risk Assessment Methods than non-health participants and were the only cohort to report workload assessment methods. Both health and non-health participants recommended that methods should be applied flexibly, and participants discussed opportunities for uplifting HF capability by teaching methods to HIT designers, particularly where access to HF experts is limited.

Our finding that non-health participants reported a wider range of Human Error Identification and Risk Assessment Methods than health participants, including workload assessment methods, is consistent with previous findings, which confirm the limited application of safety focused and workload methods in the HIT context.^19^^,^^20^ Given the high risk and potentially error prone nature of electronic medication management due to sub-optimal design, HIT design is likely to benefit from the application of safety-focused HF methods used in other sectors, especially those focused on human error identification and risk assessment.^7^^,^^20^ Similarly, with respect to workload methods, electronic health records have been linked to clinician burnout and lack of satisfaction, warranting a greater focus on the impact of system design on workload.^24^^,^^25^

Our findings shed some light on why there was limited application of error identification, risk assessment and workload assessment methods in health IT. The application of HF to healthcare including HIT is relatively recent compared to the application of HF in other industries such as aviation, transport and manufacturing^26^ and this was clear from the way non-health participants described the embedded and systematic approach they took to the application of HF methods. Use of process-type terms such as “Early Human Factors Analysis” (EHFA) and “Human Factors Integration” (HFI) by non-health experts indicates that HF practice may be more mature, structured, embedded, and advanced in non-health settings. Furthermore, there was more variability in how HF was applied by those working in health than non-health contexts who were more homogenous in their description of HF application. While an international standard related to the application of HF^18^ and some HF based guidance for the safe and usable design of health IT exists in the form of technical standards (although not to the degree that is available for and routinely applied in other safety-critical industries),^6^ our findings could suggest low awareness and routine application of these standards by HF experts working in healthcare including health IT. However, further research is needed to explore this.

We also saw more consistency in the types of methods described by health participants than non-health participants, likely reflecting a potential echo chamber effect within the health industry whereby the same subset of methods are used repeatedly.^16^^,^^20^^,^^26^ To increase adoption of a wider range of HF methods in health IT, particularly those focused on the identification of human errors and safety critical issues, we suggest further research to demonstrate the feasibility and effectiveness of the application of HF methods (or adaptations of them based on the use case) in health IT^27^ and greater knowledge translation between different sectors, possibly supported by increased movement of HF experts between sectors and transdisciplinary collaboration.^28^

While 47 methods were identified in this study, both health and non-health participants described that their decision-making process around method selection and application is guided by a framework or approach, beginning with understanding the work system and problem at hand, then selecting and flexibly applying methods based on a range of factors (Appendix C). Overall, participants stated that they initially consider factors related to the method itself such as the context of application and the method’s effectiveness in achieving the intended aim/s of application. However, this then needs to be balanced with the feasibility of applying the method within project constraints. These findings are consistent with approaches for method selection and application outlined in recognized HF textbooks.^18^^,^^29^ Our study adds to this by articulating the cognitive, rational decision-making processes employed by experienced HF experts when selecting and applying methods, an approach we recommend be adopted for HIT.

In a recent study that involved teaching HF methods to healthcare professionals to enhance patient safety practice, the upskilling of non-HF experts with appropriate HF methods demonstrated some potential.^30^ There is limited HF workforce in healthcare, with system design typically undertaken by informatics or IT personnel who are unlikely to be systematically made aware of or have exposure to HF including technical standards as part of their roles.^20^^,^^31–33^ Participants in our study recommended strategies to distill the essence of safety-focused HF methods and systems thinking (in particular, with a sociotechnical lens) to enhance their accessibility and integration into the day-to-day work of clinicians, system designers and others. In addition to teaching methods, as participants suggested, our results suggest that these efforts should also seek to support these non-HF experts in applying an approach or framework around method selection and adaptable application across the project lifecycle, akin to HF integration and decision-making processes applied by experts.^18^^,^^34^^,^^35^ Furthermore, as highlighted by participants, such guidance should promote a systems-based approach to HIT rather than solely focusing on interface design and usability focused methods, traditionally used in the field of human-computer interaction. Although many methods were identified by the participants, our complementary work has shown there is variability in method effectiveness, how easy they are to apply, and their feasibility. This highlights opportunities to further apply HF methods by HF and non-HF experts to real world projects and evaluate them.^36^ Without taking conscious efforts to uplift HF capability in the HIT context, learning from other safety critical industries and evaluating the application of HF in real world HIT projects, we may continue to see poorly designed HIT that leads to unintended consequences, including patient harm.

This study had a number of strengths such as the inclusion of both health and non-health HF experts, however, several limitations should be considered. While recruitment of participants ended once saturation was reached, the recruitment process relied on interested experts volunteering to participate in the study. This may have introduced bias in the study, and results may not be representative of all HF experts. Furthermore, the participants recruited came from several countries, sectors (eg, academia and various industries) and organizations. This study did not directly analyze the potential impact of differences in HF practices between countries, sectors and organizations, and we recommend this is a future research study. We also did not analyze differences in HF practice between participants based on individual factors such as years of experience, primary domains, project types and HF training pathways. It should also be noted that a large percentage of health HF experts had an academic background while the majority of non-health HF experts interviewed were practitioners. This may have been source of variation in the methods selected by healthcare and non-health participants. Data collection continued until thematic saturation was reached, however, the findings from the study may not be generalizable, particularly when comparing industries and roles, due to the small numbers of participants interviewed. The focus of our study was HF experts, and we did not seek advice from experts in related disciplines (eg, implementation science) who may be applying HF methods. Finally, the study explored HF methods based on self-reported perceptions and advice based on previous HIT and non-HIT projects or hypothetical HIT projects rather than objective data which can introduce recall and desirability bias. Future studies could involve the application and evaluation of HF methods to real world HIT projects through direct observation and other forms of evaluation as described in this paper. This research would be useful in generating knowledge around how safety-oriented HF methods can be applied within the HIT context to ensure that they deliver value, effectively support design and redesign, and can be feasibly integrated within the delivery of complex HIT projects which are often constrained by resources, time and budgets.

## Conclusion

By interviewing HF experts across multiple industries, this study provides insights into HF methods that can support HIT design. It identified nearly 50 HF and safety analysis methods recommended by HF experts that may be suitable for supporting the design and redesign of HIT. It also outlines a practical approach to method selection and application, as recommended by HF experts based on their practice and applied experience. Findings from this study have important implications for the design of HIT systems, and indicate that further work is required to integrate HF methods and approaches, especially those focused on safety that are used in non-health safety critical industries. Future research should involve developing practical guidance on HF application to the HIT context for system designers, and applying and evaluating HF methods within the context of real world HIT projects to demonstrate that they can be feasibly and effectively applied.

## Supplementary Material

ocag106_Supplementary_Data

## Data Availability

Data used in this study is available upon reasonable request to the corresponding author, subject to data use agreements and ethics approval.
