# Supplementary material for: Human factors methods for designing safe health information technology: what do the experts think?
Source: J Am Med Inform Assoc. 2026 Jun 29;33(10):1888–97. doi: 10.1093/jamia/ocag106 (PMC13630284; doi:10.1093/jamia/ocag106)
Supplement: Supplementary file 1 [file ocag106_supplementary_data.docx]

**Appendix A. Questions used to guide semi-structured interviews**

**Question**

Section 1: Demographics

Can you please provide a brief overview of your work in human factors including your current and previous roles?

Have you applied your human factors expertise to support the design or redesign of information technology?

Prompts:

- What kind of human factors work have you done?
- How long have you been working in human factors for?
- Please describe any work you have done around information technology or human-computer interaction

Section 2: Methods used in the design or redesign of Information Technology

Which human factors methods have you used to support the design or redesign of information technology?

*Note: if the participant is not familiar with health information technology, describe electronic medication management systems using layman’s language as an example.*

For each method, can you describe how you applied the method and provide any guidance on how others could apply the method?

Prompts:

- How would you describe the application of the method?
- What worked well and didn’t? What did you find? Would you use it again? Why?
- How would you describe the resources required to apply the method e.g. amount of time?
- Who applied the method?
- How would you describe the skill level required to apply the method?
- How would you describe the complexity and ease of applying the method?
- How would you describe the effectiveness or usefulness of the method?

Section 3: Methods for the design or redesign of electronic medication management systems

How familiar are you with electronic medication management systems (EMMS) or computerized order entry systems?

*Note: if the participant is not familiar with EMMS, provide a layman’s description of this system.*

Which human factors methods do you feel would be most appropriate for the identification of safety critical issues when designing or redesigning EMMS? Why? How would you apply them?

Section 4: General questions on method selection

How do you select human factors methods? What factors do you consider when selecting a human factors method for a particular use case or context? Why do you choose some methods over others?

Prompts:

- Characteristics of the method (e.g. ease of application, benefits/outputs, strength of evidence behind the method, cost, complexity)
- Characteristics of individuals (e.g. users applying the method including their skills, HF practice, resources, knowledge and individual beliefs)
- Internal and external contextual factors (e.g. organisational factors, project factors, culture, openness to change, external factors such as conferences)
- Implementation factors (e.g., planning needed, training requirements, champions/change agents)

How did you learn or build your knowledge around human factors methods that can be used? Where do you find information about human factors methods that can be used?

What makes a ‘good’ human factors method to you?

Which human factors methods do you feel assist most with the identification of safety critical issues? Why?

**Appendix B. Themes and sub-themes**

| **Theme 1:** Human Factors methods to support the design or redesign of Health IT  ***For each method, the following was coded:*** | |
| --- | --- |
| **Theme** | **Sub-theme** |
| Purpose of the method | To understand the work system/context/practices/tasks |
|  | To understand the problem at hand |
|  | To identify usability issues and enhance usability/design |
|  | To identify and address safety issues in the design |
| Participants | End users |
|  | Others |
| People who apply the method | Human factors specialists, ergonomists or human centred designers |
|  | Clinicians |
| Setting | Real clinical settings and work environments |
|  | Simulated settings |
| Further detail about the method | How it is applied |
| Strengths of the method | Effectiveness |
|  | Other advantages |
| Weakness of the method | Resource impost |
|  | Other disadvantages |
| **Theme 2:** Factors that influence method selection | |
| Aim of applying the method / the problem at hand | Intended outcome of using the method |
|  | The problem being solved |
|  | Where you are in the design process or project phase |
| Feasibility | Cost effectiveness |
|  | Feasibility within time and money available |
|  | Efficiency |
|  | Context of application |
|  | Project constraints |
|  | Ability to access end users |
| Effectiveness in achieving the method’s purpose | Effectiveness in identifying usability problems |
|  | Effectiveness in identifying safety issues |
|  | The ability of the method to generate useful data |
|  | Impact on the end product |
| Skillsets required | People with expertise in HF |
|  | Multidisciplinary input |
|  | Other skills required |
| Other | Method acceptability |
|  | Ability to optimise end user participation |
|  | Any other factors |
| **Theme 3:** Flexible application of methods | |
| Standard methods | Some core methods that are used regardless i.e. as a first step, use methods that help you understand the work context and problem at hand |
| Flexibility and adaptation | Application of the methods is adapted depending on resources, the project and context e.g. constraints, design/redesign |
|  | Methods or parts of methods are combined with other methods |
|  | Teaching methods or aspects of methods to non-HF experts to apply them |
| Involvement of human factors experts and application of methods at appropriate points within the project lifecycle | HF experts involved throughout the procurement, design, implementation, and evaluation lifecycle |

**Appendix C. Factors that influence method selection**

**Factor**

1. Context of application

1.1 The problem at hand e.g. issues associated with usability, safety, workload, situational awareness, interface, sociotechnical, physical, other (H, NH)
1.2 The project/product/design phase where the method/s will be applied e.g. design, redesign, configuration, optimisation, evaluation, comparative evaluation, retrospective vs predictive analysis, identify or solve problems (H, NH)
1.3 The nature of the system or component of the system e.g. standalone or integrate; the goal/intended use of the system; system complexity; whether the system is being newly designed, redesigned, or is an off the shelf system being configured (H, NH)
1.4 The required domain areas/constructs to be covered (e.g. usability, safety, workload, situational awareness, decision making) (NH)

2. Effectiveness of the method in achieving the aim of its application

2.1 Method’s effectiveness in understanding the dynamic nature of the system (NH)

2.2 Whether the method supports observing work as done rather than work as imagined (NH)

2.3 Method’s effectiveness in identifying HF issues e.g. usability problems (H, NH)

2.4 Method’s effectiveness in improving user experience (H, NH)

2.5 Method’s ability to generate usable and useful results/actionable insights (H, NH)

2.6 Whether the method is validated and/or produces outcomes that align with gold standard (H)

2.7 The method’s reliability if applied by different people (H)

3. Feasibility of applying the method

3.1 Cost effectiveness (H, NH)

3.2 Feasibility within time and money available (H, NH)

3.3 Efficiency (H, NH)

3.4 Ability to access end users/participants and their ability to participate (H, NH)

3.5 Skills, expertise and experience of the person/s applying the method (H, NH)

3.6 Availability of data and inputs required to enable method application (NH)

3.7 Location and setting in which the method will be applied e.g. remote vs onsite (H)

3.8 Do-ability and ease of use (H)

3.9 Ability to optimise end user participation and make it easy for them to participate e.g. combining with another method that involves users (H)

4. Other

4.1 The industry in which the work is being done (NH)

4.2 Other disciplines contributing to the project (NH)

4.3 Information already available (NH)

4.4 Operating assumptions (NH)

4.5 Micro, meso and macro level considerations that have implications on the safety of the design (H)

4.6 Method acceptability by different users and the hospital (H)

4.7 Ability to use the same method with multiple user groups / the method’s ability to accommodate different perspectives (H)

4.8 How innovative the method is (H)

4.9 The roles of the human factors experts involved in a project i.e. designers and/or evaluators

4.10 Method’s ability to engage users to participate (H)

4.11 Method’s ability to enable effective communication between different user groups/ project team members (H)
